# The survival of childhood leukemia: An 8‐year single‐center experience

**DOI:** 10.1002/cnr2.1784

**Published:** 2023-01-26

**Authors:** Mohammadreza Bordbar, Nazila Jam, Mehran Karimi, Mahdi Shahriari, Soheila Zareifar, Omid Reza Zekavat, Sezaneh Haghpanah, Hadi Mottaghipisheh

**Affiliations:** ^1^ Hematology Research Center Shiraz University of Medical Sciences Shiraz Iran; ^2^ Pediatrics Department, Medical School Shiraz University of Medical Sciences Shiraz Iran

**Keywords:** children, hepatomegaly, leukemia, survival

## Abstract

**Background:**

The survival of childhood leukemia has improved. We aimed to report the survival rate and the associated factors in children with acute leukemia during an 8‐year follow‐up.

**Aims:**

This study investigates the 8‐year survival rates of children with acute myeloid leukemia (AML) and acute lymphoblastic leukemia (ALL) in Shiraz, the largest oncology center in Southern Iran. We also aimed to assess the independent factors associated with higher mortality in childhood leukemia.

**Methods:**

Children 0–18 years with acute leukemia were followed from 2013 to 2021 in Shiraz, Iran. The 8‐year overall survival (OS) and event‐free survival (EFS) rates were estimated by the Kaplan–Meier method. Independent factors associated with survival were assessed by the Cox regression hazard modeling.

**Results:**

We included 786 children, with 43.5% female, and a mean age of 6.32 ± 4.62 years. Patients with AML compared to ALL experienced more relapse (34.6% vs. 22.5%, *p* = .01) and death (31.7% vs. 11.3%, *p* < .001). The cumulative 8‐year OS and EFS were 81% (95% confidence interval (CI), 74.3% to 86.1%) and 68.3% (95% CI, 63.5% to 72.7%) in ALL patients and 63.5% (95% CI, 52.1% to 72.9%) and 43% (95% CI, 33.1% to 52.6%) in AML patients. Multivariable analysis revealed that hepatomegaly (hazard ratio = 4, 95% CI, 1.0 to 22.3, *p* = .05) was the main independent risk factor of death in ALL patients. No definite risk factor was defined for AML patients.

**Conclusion:**

The survival of childhood leukemia has recently increased dramatically in low‐middle income countries. Hepatomegaly was introduced as a potential risk factor for lower survival in ALL patients. Further multicenter studies are needed to confirm the validity of this association.

## 1 INTRODUCTION

Acute leukemia accounts for approximately 30% of all childhood malignancies and is the most common cancer in children. Acute lymphoblastic leukemia (ALL) is five times more common in children than acute myeloid leukemia (AML).^1^ The outcomes for ALL have steadily improved since the 1980s.^2^ Five‐year event‐free survival (EFS) for childhood ALL approaches 90% in developed countries.^3^, ^4^ Five‐year and estimated 10‐year overall survival (OS) rates were reported as 90% and 88%, respectively.^5^ In contrast, cure rates are less than 35% in the developing world, in part because of abandonment of treatment and lack of dedicated, multidisciplinary pediatric oncology units.^6^, ^7^, ^8^ When children in the developing world are treated with standard international protocols, 5‐year EFS and OS rates are improved (74% and 82%, respectively). Yet, treatment‐related mortality (TRM) remains higher than in the developed world (5% death in complete remission [CR]).^9^, ^10^ Similarly, survival rates for AML have greatly improved over the past several decades; however, OS for children with AML is approximately 65% to 70% and remains lower than for children with ALL.^11^

Iran is one of the developing countries with a high prevalence of leukemia, with an age‐standardized rate of 7.7 in men and 5.5 in females per 100 000 persons‐years.^12^ Some studies have reported a cumulative 5‐year survival rate of 43%–53%.^13^ This study investigates the 8‐year survival rates of children with AML and ALL in Shiraz, the largest oncology center in Southern Iran. We also aimed to assess the independent factors associated with higher mortality in childhood leukemia.

## 2 METHODS

This historical cohort study from 2013 to 2021 included pediatric patients aged 0–18 years with acute leukemia admitted to Amir Oncology Hospital affiliated with Shiraz University of Medical Sciences. Children who did not complete their treatment or had lost to follow‐up were excluded from the study. The leukemia diagnosis was confirmed in bone marrow (BM) if it contained more than 25% blast. The subtype of leukemia was defined by flow cytometry including pre‐B cell ALL, T‐cell ALL, AML‐M3, AML‐non M3, and undifferentiated or mixed‐phenotype acute leukemia (MPAL). The demographic data, initial laboratory data, and clinical presentations were recorded. EFS was calculated from the date of diagnosis to the last follow‐up or first event (relapse or death). OS was calculated from the date of diagnosis to death of any cause. The Ethics Committee of Shiraz University of Medical Sciences approved the study with code number IR.SUMS.MED.REC.1399.358.

### 2.1 Treatment protocols

Patients with ALL were treated based on their risk group stratification. The induction of remission in the standard group (age 1–10 years, WBC < 50 × 10^9^/L, no cerebral spinal fluid (CSF) involvement, and favorable cytogenetic study) included weekly vincristine for 4 weeks, pegasparaginase on day 4, dexamethasone for 28 days, and intrathecal injection of methotrexate on days 1, and 15. BM assessment with flow cytometry was done on day 15 for early response, and on day 29 to determine BM remission. Treatment was intensified in patients with a slow early response (BM blast on day 15 ≥ 5% by morphology) or positive end‐of‐induction (EOI) minimal residual disease (MRD) (BM MRD ≥0.01% on day 29; available very recently in our center with very limited data). In high‐risk patients, 4 courses of daunorubicin were added to the treatment protocol. Those who achieved complete remission continued their treatment in the consolidation, interim maintenance, delayed intensification, and maintenance phases of treatment. Patients with poor response to chemotherapy, induction failure, early BM relapse (less than 18 months from diagnosis) or recurrent BM or central nervous system (CNS) relapses, and unfavorable cytogenetics including Philadelphia chromosome were planned for hematopoietic stem cell transplantation (HSC) from every available full‐matched donor.^14^ For those without a full‐matched donor, haploidentical hematopoietic stem cell transplantation (HSCT) from their parents was suggested. Those with late BM relapse (more than 18 months from diagnosis) and infant leukemia were planned for HSCT only if a full‐matched donor was found.

Patients with AML were treated according to the *AML* Berlin‐Frankfurt‐Munster (*BFM*)‐2012 protocol.^15^ Patients with unfavorable cytogenetics were treated as a high‐risk group and were candidates for HSCT in the first remission. Patients with relapsed/refractory AML were treated with salvage protocols such as FLAG‐Ida and then HSCT as soon as they went into remission.

### 2.2 Statistical analysis

OS and EFS at 8 years were estimated using the Kaplan–Meier method and were compared by the Log‐Rank test. Prognostic factors considered in the univariate analysis for ALL included age, gender, initial clinical signs at diagnosis, abnormal laboratory findings, blast counts on days 15 and 29 of induction, CSF status at diagnosis, and BM or CNS relapse. In patients with AML, BM blast counts after the first and second inductions, and abnormal clinical and laboratory data were included. As there was a lot of missing data in the results of cytogenetics, they were not included in the univariate analysis. Similarly, the data on EOI MRD by multicolor flow cytometry was very limited, because this technique was very recently available in our center, so a few patients were assessed with MRD. Therefore, MRD data was not entered to correlate with the outcomes. The variables with a *p* value less than .2 were entered in the multivariable analysis. Cox proportional hazard modeling was used to assess the effect of prognostic factors on survival. Events were defined as death or disease relapse. The results were reported as a hazard ratio (HR) with a 95% confidence interval (CI). A *p* value less than .05 was considered statistically significant.

## 3 RESULTS

This study included 786 patients with acute leukemia, 43.5% female, with a mean age of 6.32 ± 4.62 years. The study subgroups included 585 (74.4%) cases of pre‐B ALL, 94 (12%) patients with T‐ALL, 88 (11.2%) with AML‐non M3, 16 (2%) with AML‐M3, and 3 (0.4%) patients with undifferentiated or MPAL. Most of the patients were in the age range of 1–10 years in ALL and AML subgroups. Table 1 shows the demographic data and initial clinical and laboratory findings of the study population. Clinical and laboratory findings were comparable in ALL and AML patients except for ecchymosis, bleeding, and neurologic and gastrointestinal symptoms, which were more frequently seen in AML patients.

**TABLE 1:** Demographic, clinical, and laboratory data of the study population.

	ALL (*n* = 679)	AML (*n* = 104)	*p*‐value
Age (year) Median (range)	5 (0.25–18)	7 (0.16–17)	
Age category, n (%)			
<1 year	58 (8.5)	16 (15.4)	<.001
1‐ < 10 year	492 (72.6)	46 (44.2)	
≥ 10 year	128 (18.9)	42 (40.4)	
Gender (F), n (%)	292 (43)	49 (47.1)	.46
*Initial clinical findings*			
Hepatomegaly, n (%)	266 (31.2)	23 (22.3)	.08
Splenomegaly, n (%)	824 (33)	30 (28.8)	.43
Lymphadenopathy, n (%)	71 (10.5)	5 (4.8)	.07
Bone pain, n (%)	34 (5)	2 (1.9)	.21
Anorexia, n (%)	61 (9)	8 (7.7)	.72
Ecchymosis, n (%)	29 (4.3)	12 (11.5)	.004
Bleeding, n (%)	18 (2.7)	13 (12.5)	<.001
Headache, n (%)	15 (2.2)	3 (2.4)	.72
Respiratory symptoms, n (%) (cough, dyspnea,…)	88 (13)	13 (12.5)	>.999
Neurologic symptoms, n(%)	13 (1.9)	7 (6.7)	.01
Gastrointestinal symptoms, n (%) (nausea, vomiting, diarrhea, …)	25 (3.7)	11 (10.7)	.004
Abdominal pain, n (%)	47 (6.9)	6 (5.8)	.69
*Initial lab findings*			
WBC (10^9^/L) median (range)	9.1 (0.1–779.0)	16.4 (0.2–263.8)	
Hemoglobin (g/dL) median (range)	7.9 (2.5–14.8)	8.1 (3.5–12.6)	
Platelet (10^9^/L) median (range)	51 (4–721)	33 (6–339)	
LDH (mg/dL) median (range)	621 (156–18 200)	726.5 (177–11 700)	
Uric acid (mg/dL) median (range)	3.5 (0.4–36)	3.6 (1.1–8.7)	
ESR (mm/hour) median (range)	52 (2–150)	64.5 (2–140)	
WBC > 10 × 10^9^/L, n (%)	314 (46.9)	59 (56.7)	.057
WBC (10^9^/L), n (%)			.24
10–50	193 (28.8)	38 (36.9)	
50–100	46 (6.9)	7 (6.8)	
>100	75 (11.2)	14 (13.6)	
Low hemoglobin^a^	631 (94.3)	96 (93.2)	.82
Platelet <150 × 10^9^/L, n(%)	542 (81)	89 (86.4)	.22
LDH ≥500 mg/dL, n(%)	443 (66.6)	74 (72.5)	.26
Uric acid ≥6 mg/dL, n (%)	108 (16.2)	11 (10.7)	.19
ESR≥20 mm/hour. N (%)	415 (79.8)	74 (86)	.19

Abbreviations: ALL, acute lymphoblastic leukemia; AML, acute myeloid leukemia; ESR, erythrocyte sedimentation rate; LDH, lactic dehydrogenase; WBC, white blood cell.

^a^ Low hemoglobin (<10.5 g/dL for age <2 years; <11.5 g/dL for age 2–12 years; <12 g/dL for age 12–18 years).

**FIGURE 1:**
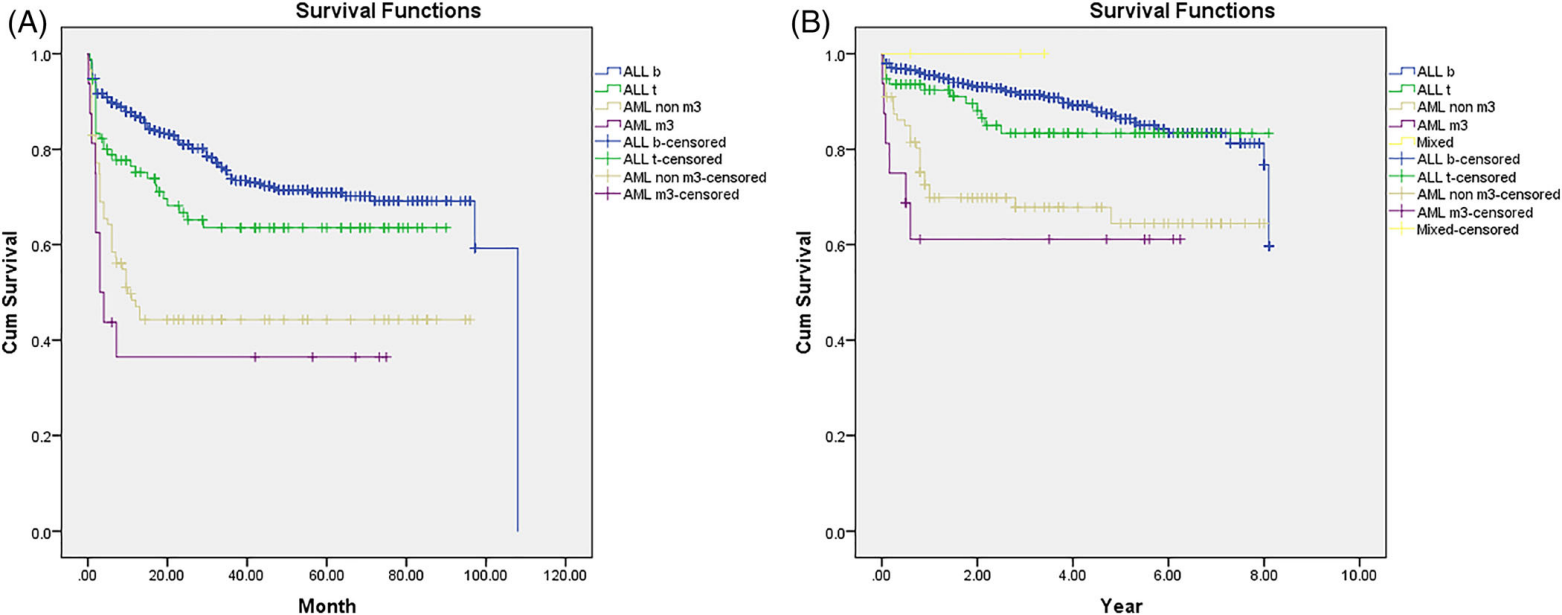
Kaplan–Meier survival curve of 8‐year even‐free survival (1A) and overall survival (1B) in children with acute leukemia (*P* < .001).

Patients with AML compared to ALL experienced more relapse (34.6% vs. 22.5%, *p* = .01) and death (31.7% vs. 11.3%, *p* < .001). About two‐thirds of the patients who experienced disease relapse survived both in ALL and AML groups. There was no death in the three patients with MPAL. Among patients with a diagnosis of ALL and AML, the best survival was observed in pre‐B ALL immunophenotype while patients with AML‐M3 had the worst prognosis (Table 2). The mean survival (95% CI) was 7.18 (6.98 to 7.38) years in ALL and 5.44 (4.73 to 6.16) years in AML patients (*p* < .001). The cumulative 5‐ and 8‐year OS were 86.4% (95% CI, 82.8% to 89.3%) and 81% (95% CI, 74.3% to 86.1%) in ALL patients and 63.5% (95% CI, 52.1% to 72.9%) in AML patients (Figure 1B). The mean (95% CI) EFS in ALL and AML patients were 78.8 (74.8 to 83.0) months and 43.7 (34.7 to 52.8) months, respectively (*p* < .001). The cumulative 5‐ and 8‐year EFS were 69.9% (95% CI, 65.5% to 73.8%) and 68.3% (95% CI, 63.5% to 72.7%) in ALL and 43% (95% CI, 33.1% to 52.6%) in AML patients (Figure 1A).

**TABLE 2:** Overall and event‐free survival in the study subgroups.

Immunophenotype	Deaths, n (%)	Overall survival time (months) mean (95% CI)	*p*‐value	Events	Event‐free survival time (months) mean (95% CI)	*p*‐value
Pre‐B ALL	64 (10.9)	86.5 (83.9–89.1)	<.001	147 (25.1)	79.9 (75.6–84.3)	<.001
T‐ALL	13 (13.8)	83.4 (76.5–90.3)		33 (35.1)	60.4 (51.8–69.0)	
AML‐non M3	27 (30.7)	66.2 (56.9–75.5)		47 (53.4)	45.0 (35.2–54.8)	
AML‐M3	6 (37.5)	46.9 (29.3–64.7)		10 (62.5)	28.9 (11.5–46.4)	
MPAL	0 (0)	‐		0 (0)	‐	

Abbreviations: ALL, acute lymphoblastic leukemia; AML, acute myeloid leukemia; CI, confidence interval; MPAL, mixed‐phenotype acute leukemia.

The Log‐Rank test analyzed the effects of different variables on survival. Among the included variables in ALL patients, BM or CNS relapse, initial WBC counts ≥50 × 10^9^/L, hepatomegaly, uric acid ≥6 mg/dL, and CSF involvement at diagnosis were associated with worse survival. Regarding AML patients, initial WBC counts ≥100 × 10^9^/L, and erythrocyte sedimentation rate (ESR) < 20 mm/h were associated with lower survival rates (Table 3).

**TABLE 3:** Univariate analysis of the variables affecting survival in patients with acute leukemia.

Variables	Mean survival (year) (95% CI)	*p*‐value
**Acute lymphoblastic leukemia**		
*Hepatomegaly*		
Yes	6.9 (6.5 to 7.3)	.009
No	7.4 (7.2 to 7.7)	
*Relapse*		
Yes	6.0 (5.5 to 6.5)	<.001
No	7.7 (7.5 to 7.9)	
*BM blast on day 15*		
≥5%	7.3 (6.9 to 7.7)	.346
<5%	7.5 (7.1 to 7.9)	
BM blast on day 29		
≥5%	4.2 (3.2 to 5.2)	.052
<5%	6.8 (5.9 to 7.8)	
WBC (10^9^/L)		
≥50	6.6 (6.0 to 7.2)	.005
<50	7.4 (7.2 to 7.6)	
Platelet (10^9^/L)		
≥150	7.6 (7.3 to 8.0)	.064
<150	7.2 (7.0 to 7.4)	
Uric acid (mg/dL)		
≥6	6.9 (6.4 to 7.4)	.044
<6	7.3 (7.1 to 7.6)	
LDH (mg/dL)		
≥500	7.1 (6.9 to 7.4)	.066
<500	7.6 (7.3 to 7.9)	
Age at diagnosis (Y)		
<1	6.6 (5.8 to 7.4)	.071
1‐ < 10	7.3 (7.1 to 7.5)	
≥10	6.9 (6.4 to 7.4)	
CSF status at diagnosis		
Involved	4.9 (2.5–7.3)	<.001
Not involved	7.2 (7.0–7.4)	
**Acute myeloid leukemia**		
*Hepatomegaly*		
Yes	4.1 (2.7 to 5.8)	.165
No	5.8 (5.0 to 6.6)	
*WBC (10* ^*9*^ */L)*		
≥100	2.5 (0.9 to 3.9)	.001
<100	5.9 (5.2 to 6.6)	
*ESR (mm/h)*		
≥20	6.0 (5.2 to 6.8)	.038
<20	2.7 (1.1 to 4.2)	
*Uric acid (mg/dL)*		
≥6	3.1 (1.4 to 4.8)	.077
<6	5.7 (5.0 to 6.5)	
*LDH (mg/dL)*		
≥500	5.1 (4.3 to 6.0)	.134
<500	6.2 (5.1 to 7.3)	
*End of 1st induction BM blast*		
≥5%	5.6 (4.5 to 6.8)	.614
<5%	4.9 (3.1 to 6.6)	
*End of 2nd induction BM blast*		
≥5%	1.8 (0.0 to 4.2)	.091
<5%	4.7 (3.8 to 5.7)	

Abbreviations: BM, bone marrow; CI, confidence interval; ESR, erythrocyte sedimentation rate; LDH, lactic dehydrogenase; WBC, white blood cell.

Multivariable analysis with Cox regression modeling revealed that hepatomegaly increased the risk of death by almost 5 times in ALL patients (HR = 4.7, 95% CI, 1.0 to 22.3). Other variables including relapse, WBC counts ≥50 × 10^9^/L, LDH ≥500 mg/dL, and EOI BM blast ≥5% were associated with poor outcomes but the estimates had wide CIs(Table 4). Regarding AML patients, ESR < 20 mm/h increased the risk of death by 10%. Again the risk estimate had a wide CI (HR = 1.1, 95% CI, 0.1 to 13.9).

**TABLE 4:** Cox regression multivariable analysis of independent variables affecting survival in patients with acute leukemia.

Variables	Hazard ratio (95% confidence interval)
*Acute lymphoblastic leukemia*	
Hepatomegaly	4.7 (1.0 to 22.3)
Relapse	2.7 (0.7 to 10.9)
BM blast on day 29	1.9 (0.4 to 9.3)
WBC counts ≥ 50 × 10^9^/L	1.7 (0.18 to 15.9)
Uric acid ≥ 6 (mg/dL)	1.9 (0.2 to 15.0)
LDH ≥ 500 (mg/dL)	2.4 (0.4 to 12.4)
*Acute myeloid leukemia*	
ESR < 20 (mm/h)	1.1 (0.1 to 13.9)

Abbreviations: BM, bone marrow; LDH, lactic dehydrogenase; WBC, white blood cell.

## 4 DISCUSSION

This cohort study reviewed the demographic and clinical presentations of children with acute leukemia in a referral center in Southern Iran. We also reported the OS, EFS, and the related associated factors during an eight‐year follow‐up period. As expected, pre‐B ALL comprised the most common diagnosis in ALL patients (85%) and the entire cohort. The age at diagnosis was higher in AML than in ALL patients. Although most of the patients in both groups were younger than 10 years, the proportion of patients who were older than 10 years was higher in AML than in ALL subgroups (40.4% vs. 18.9%). Hepatosplenomegaly and lymphadenopathy as the initial clinical presentation were more frequently observed in ALL than in AML, though the difference was not statistically significant. It seems that lymphoblasts tend to infiltrate extramedullary organs much sooner than myeloblasts. On the other hand, bleeding and ecchymosis were more frequently observed in patients with AML compared to ALL. Coagulopathy and lower platelet counts in AML patients may explain the difference.

Patients with AML experienced more relapses than ALL and consequently lower survival rates. Relapse is a major risk factor determining the outcome of patients with leukemia, which in part is affected by the residual burden of the disease or MRD.^16^ The relapse rate in ALL was reported to be around 20% in previous studies with an extremely poor prognosis manifested as a 5‐year OS of about 20%–30%. The site of relapse, time to relapse, and cytogenetic aberrations determine the prognosis of children with relapse.^17^ Similarly, we observed a relapse rate of 22.5% in ALL patients. They showed an inferior outcome with about 3 times the risk of death compared to non‐relapsed patients.

The cumulative 5‐year OS was 86.4% (95% CI, 82.8% to 89.3%) in our patients with ALL. The outcome of children with ALL has greatly improved in recent years due to the advent of novel treatment regimens, the introduction of target therapies, and better monitoring of treatment response with MRD status. The 5‐year OS has increased from 57% in the 1970 s up to 96% in most recent studies.^18^, ^19^, ^20^, ^21^ However, the outcome of childhood ALL in low‐middle income countries is still suboptimal with a 5‐year OS approaching 50%–70% in most reports.^16^, ^17^, ^22^, ^23^ A meta‐analysis including 10 studies between the years 2002 and 2015 in Iran reported a 5‐year OS of 71% (95% CI, 68% to 74%) in ALL and 46% in AML patients.^24^ Similarly, two previous reports in our center during 1995–2000 and 2004–2009 showed a 5‐year OS of 72.5% and 76.9% in children with ALL.^25^, ^26^ The results imply that the outcome of childhood ALL has improved dramatically in our center compared to previous years and in other parts of the country. The main reason for this improvement may be the updated treatment regimens, monitoring of MRD with multicolor flow cytometry, and better supportive care reducing non‐relapse mortality.

Furthermore, the outcome of children with AML has improved in developed countries with an OS of about 70% in most reports. This improvement in survival is mainly attributed to risk‐adapted intensive chemotherapy, HSCT, and better supportive care.^27^, ^28^ However, in resource‐limited countries, survival is still much lower with OS and EFS below 40%. Factors such as late presentation, malnutrition, and TRM were reported to be contributing.^29^ We encountered a much better outcome in our AML patients, with an 8‐year OS of 63.5%, which was very close to the statistics of developed countries. The 8‐year EFS was 43%, which is mostly attributed to the high relapse rate in childhood AML despite intensive chemotherapy. Early HSCT after CR1 may decrease the chance of relapse and improve their outcome. Interestingly, the 5‐ and 8‐year OS and EFS were similar in our patients with AML. It means that no death or relapse occurred in the survivors of childhood AML 5‐years following treatment completion.

Univariate analysis revealed that relapse, hepatomegaly, CSF involvement, WBC count >50 × 10^9^/L at diagnosis, and abnormal uric acid were associated with inferior outcomes in patients with ALL. EOI BM blast ≥5% was also associated with lower survival, but with marginal significance. Multivariable Cox regression analysis confirmed that hepatomegaly remained to be an independent factor associated with worse OS. Traditionally, age ≥ 10 years, WBC count ≥50 × 10^9^/L, unfavorable cytogenetics, and treatment response were the main prognostic factors in patients with ALL.^30^ It seems that MRD is the strongest predictor of outcome in patients with ALL. It combines the interaction of factors such as genetic subtypes, the effects of chemotherapy drugs, and host genetics that affect drug metabolism.^31^ MRD is especially important in patients with T‐cell ALL in which other prognostic factors such as age and WBC counts lose their values.^32^ Previous reports in Iran highlighted the impact of relapse and high WBC counts at diagnosis (≥ 50 × 10^9^/L) on survival in childhood ALL.^13^, ^24^ We also showed that relapse was associated with about three times the risk of death in children with ALL, though the certainty of the effect was weakened by the wide CI.

Hepatomegaly is a common manifestation in ALL that is seen in up to 50% of patients.^33^ Acute leukemia may present with acute liver failure which adversely affects the outcome of treatment.^34^, ^35^ Hepatomegaly was also reported to associate with hyperleukocytosis and a higher rate of death in adults with chronic myeloid leukemia in the chronic phase.^36^ However, The association between hepatomegaly and worse outcomes in acute leukemia was never reported previously. We reported for the first time that hepatomegaly is associated with about five times higher risk of mortality in children with ALL. It may indicate the likelihood of extramedullary dissemination of blats which in turn increases the risk of treatment failure and disease recurrence.^37^

We also observed that normal ESR at diagnosis was associated with 10% higher mortality, though the estimate had a wide CI which makes the assumption less certain. High ESR is a non‐specific sign of inflammation that is commonly observed in childhood leukemia. If a patient with leukemia has normal ESR, it may indicate the rapid progression of the illness and insufficient time to raise ESR. The same concept is relevant in children with normal hemoglobin levels at presentation which was found to be associated with lower OS in AML.^29^ Other variables such as high WBC counts, malnutrition, unfavorable cytogenetics, resistant disease to induction chemotherapy, and high body mass index were reported to correlate with poor outcomes in some reports.^29^, ^38^

Our study had some limitations and strengths. We reported the outcome of a relatively large number of children with acute leukemia during a long follow‐up period. We could introduce some novel risk factors for death, which merit further investigation in future studies.

However, the study faced some limitations. First, the data on cytogenetic abnormalities were missing and could not be analyzed in the Cox regression modeling. Second, MRD was reported by morphologic assessment of BM smears and not by novel techniques such as multicolor flow cytometry or real‐time polymerase chain reaction. Finally, the wide confidence intervals for the HRs of lower survival may affect the certainty with which we may conclude that the mentioned variables are truly risk factors. Repeating the analyses with larger sample sizes may serve as a solution.

### 4.1 Conclusion

The survival of childhood leukemia has recently increased dramatically in low‐middle‐income countries. Hepatomegaly is the main risk factor for death in ALL patients. EOI positive MRD, relapse, hyperleukocytosis, abnormal LDH and uric acid, and normal ESR at diagnosis are other potential contributing factors to lower survival. Further multicenter studies are needed to confirm the validity of this association.

## AUTHOR CONTRIBUTIONS

**Mohammad Reza Bordbar:** Conceptualization (equal); project administration (equal); supervision (equal). **Nazila Jam:** Data curation (equal); resources (equal); writing – review and editing (equal). **Mehran Karimi:** Resources (equal); writing – review and editing (equal). **mahdi Shahriari:** Resources (equal); writing – review and editing (equal). **Soheila zareifar:** Resources (equal); writing – review and editing (equal). **Omid Reza Zekavat:** Resources (equal); writing – review and editing (equal). **Sezaneh Haghpanah:** Formal analysis (equal); software (equal). **Hadi Mottaghipisheh:** Investigation (equal); methodology (equal); writing – original draft (equal); writing – review and editing (equal).

## CONFLICT OF INTEREST STATEMENT

The authors have stated explicitly that there are no conflicts of interest in connection with this article.

## ETHICS STATEMENT

This study was approved by the Shiraz University of Medical Sciences Local Ethics Committee (code: IR.SUMS.MED.REC.1399.358). The study protocol conformed to the ethical guidelines of the 1975 Helsinki Declaration. All participants signed a written informed consent before sampling. The study protocol conformed to the ethical guidelines of the 1975 Helsinki Declaration. Written informed consent was obtained from all subjects and/or their legal guardian(s).

## Data Availability

The datasets generated and/or analyzed during the current study are not publicly available due but are available from the corresponding author on reasonable request.
